# Estradiol—PGC‐1 signaling in mitochondrial health: Implications for perimenopause and cardiovascular disease

**DOI:** 10.14814/phy2.71155

**Published:** 2026-10-21

**Authors:** Alyssa L. Parten, Glenn C. Rowe

**Affiliations:** ^1^ Division of Cardiovascular Disease, Department of Medicine University of Alabama at Birmingham Birmingham Alabama USA

**Keywords:** cardiovascular, estradiol, estrogen, menopause, mitochondria, PGC‐1

## Abstract

Cardiovascular disease (CVD) is the leading cause of death in aging women. While CVD etiology is multifactorial, mitochondrial dysfunction, characterized by reduced content, impaired energy production, and increased oxidative stress, is an underlying contributor. These impairments reduce the ability of the heart and vasculature to support peripheral tissues. Estradiol, a key sex hormone with widespread physiological effects, supports mitochondrial function, lipid oxidation, vascular endothelial cell and smooth muscle function, and redox balance, contributing to its cardioprotective effects in women. Perimenopause is marked by hormonal variability and an overall decline in estradiol concentrations that may compromise mitochondrial quality and vascular function, increasing CVD risk. Mitochondrial quality and maintenance are largely controlled by the peroxisome proliferator‐activated receptor gamma coactivator‐1 (PGC‐1) family of transcriptional coactivators. Impairments in PGC‐1 isoform expression have been linked to cardiovascular and metabolic decline and may be exacerbated by estradiol loss during the menopause transition. However, the mechanisms by which estradiol signaling regulates PGC‐1 isoforms, and how this interaction contributes to mitochondrial dysfunction and CVD development following menopause, have not been comprehensively described. Thus, this review aims to highlight current evidence on the individual and overlapping roles of estradiol and PGC‐1 isoforms and examine how their interaction may contribute to the development of CVD following menopause.

## 1 INTRODUCTION

Female‐specific cardiovascular prevention guidelines were not introduced until 1999 (Mosca et al., 1999, 2007), and only in the early 2000s was cardiovascular disease (CVD) broadly acknowledged as a leading cause of morbidity and mortality in women (Elias‐Smale et al., 2015). Since then, growing evidence has demonstrated that CVD not only presents differently in women compared to men, but that risk increases substantially following menopause (Mosca et al., 2007). Population‐based data indicate that natural menopause occurs on average at 50 years of age (Pasokh et al., 2024). Prior to this transition, women maintain a 50% lower risk of CVD than age‐matched men (Mosca et al., 2007). Perimenopause, defined by fluctuating ovarian hormone production preceding the permanent cessation of menses and ovulation, represents a critical transition between the premenopausal and postmenopausal stages, during which CVD risk begins to rise (Figure 1) (Mosca et al., 2007). Despite the multifactorial etiology of CVD, at the ‘heart’ of this pathological condition is mitochondrial dysfunction. Mitochondrial dysfunction reflects a deficit in the organelles’ ability to meet cellular energetic demands and manifests from reduced oxidative phosphorylation (OXPHOS) activity, impaired adenosine triphosphate (ATP) synthesis, and elevated reactive oxygen species (ROS) (Zong et al., 2024). These effects on mitochondrial quality implicate multiple physiological systems, including metabolic, muscular, and cardiovascular (Zong et al., 2024). Within the vasculature, mitochondrial dysfunction can increase ROS production, reacting to nitric oxide (NO) and reducing its bioavailability; thereby, impairing endothelial nitric oxide synthase (eNOS)‐stimulated vasodilation (Zong et al., 2024). The downstream consequences of these mitochondrial disturbances may then include endothelial dysfunction, impaired blood flow, and an increase in cardiovascular strain—all of which contribute to a pathological cycle that increases long‐term CVD risk (Gutierrez‐Huerta et al., 2025).

**FIGURE 1:**
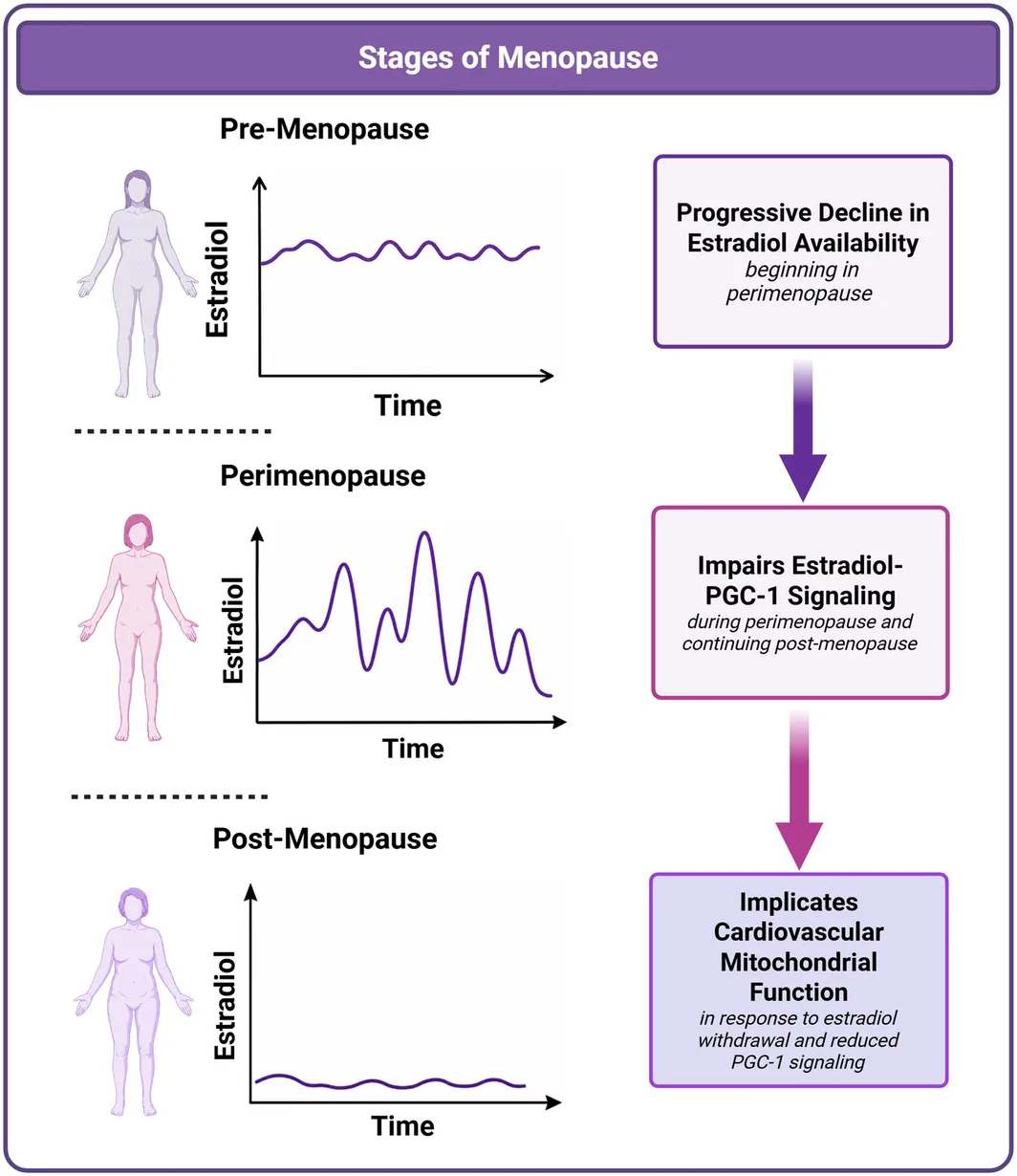
Changes in estradiol availability across the stages of menopause and proposed implications for PGC‐1 signaling and cardiovascular mitochondrial function. *Pre‐menopause*: Estradiol concentrations are relatively predictable fluctuations that regulate the menstrual cycle and ovarian function. Cyclical estradiol availability supports PGC‐1 function and associated cardiovascular mitochondrial processes. *Perimenopause*: Increasingly variable followed by declining estradiol fluctuations that accompany perimenopause may begin to impair estradiol‐PGC‐1 pathways and their associated effects on cardiovascular mitochondrial function. *Post‐menopause*: Following 12 consecutive months without menses, estradiol remains chronically low. Sustained estradiol loss may further reduce PGC‐1 activity, further compromising cardiovascular mitochondrial function, potentially contributing to increased cardiovascular risk.

Mitochondrial quality of skeletal muscle, cardiac muscle, and the vascular wall (i.e., endothelial and vascular smooth muscle cells) are largely regulated by the peroxisome proliferator‐activated receptor γ coactivator (PGC‐1) family of transcriptional coactivators (PGC‐1α, PGC‐1β, and PGC‐1‐related coactivator [PRC]) (Qian, Zhu, et al., 2024). Of the isoforms, PGC‐1α is widely recognized as a master regulator of mitochondrial biogenesis, while PGC‐1β and PRC have overlapping roles in regulating mitochondrial function (Qian, Zhu, et al., 2024). Evidence to date indicates that reductions in PGC‐1α and PGC‐1β expression compromise mitochondrial function, resulting in impaired bioenergetic capacity, reduced oxidative stress resilience, and blunted adaptive responses (Zong et al., 2024). Conversely, in experimental models, moderate increases in PGC‐1α and PGC‐1β activity enhance mitochondrial biogenesis and respiration, thereby improving mitochondrial quality (Lehman et al., 2000; Srivastava et al., 2009). In cardiac muscle, forced PGC‐1α protein expression has been shown to increase mitochondrial biogenesis, OXPHOS gene expression, fatty acid oxidation, and coupled respiration (Lehman et al., 2000), whereas a loss of PGC‐1α in mechanistic models results in the opposite phenotype and impairs cardiac and metabolic responses (Leone et al., 2005). These findings indicate that PGC‐1 coactivators govern mitochondrial adaptation, such that reductions in their protein expression and coactivator activity increase susceptibility to mitochondrial dysfunction and related chronic diseases (Qian, Zhu, et al., 2024).

Estradiol has been widely described as “cardioprotective” due to its systemic interactions with the cardiovascular system (Iorga et al., 2017). Estradiol has been shown to increase eNOS activity and NO‐mediated vasodilation (Haynes et al., 2000), enhance antioxidant and anti‐inflammatory defenses (Kemper et al., 2014), and promote angiogenesis and anti‐atherogenic signaling (Caliceti et al., 2013). The menopausal rise in cardiovascular risk coincides with declining estradiol availability, implicating the loss of these protective processes in subsequent health decline. In addition, PGC‐1α appears to be sensitive to changes in estradiol availability, with low estradiol associated with reduced PGC‐1α protein expression and subsequent mitochondrial function (Kemper et al., 2014; Park et al., 2017; Tian et al., 2025). In both rodent and human models, estradiol treatment or estradiol‐containing hormone therapy has been reported to augment PGC‐1α protein expression or nuclear abundance and improve related markers of mitochondrial content and function (Kleis‐Olsen et al., 2024; Park et al., 2017; Tian et al., 2025). Women within 6 years post‐menopause have shown increased nuclear skeletal muscle PGC‐1α abundance and AMPK phosphorylation with short‐term, high‐dose transdermal 17β‐estradiol administration (Park et al., 2017). Furthermore, long‐term combined estradiol‐progestin therapy was associated with greater skeletal muscle mitochondrial content and respiratory capacity, as well as lower visceral and total fat mass, compared with matched nonusers (Kleis‐Olsen et al., 2024). This evidence supports a potential relationship between estradiol signaling, PGC‐1α regulation, and the maintenance of mitochondrial and cardiometabolic health in women.

Given the high prevalence of CVD among aging women, declining estradiol availability may reduce PGC‐1 isoform activity and thereby contribute to CVD development. Previous reviews have detailed the relationships between declining estradiol, mitochondrial dysfunction, and cardiovascular outcomes, as well as implications of reduced PGC‐1 isoform activity for these processes (Huang et al., 2025; Rowe et al., 2010). The present review synthesizes research connecting estradiol availability to PGC‐1 isoform expression and coactivator activity, with key studies from the past 10 years summarized in Table 1. The review aims to elucidate potential mechanisms underlying mitochondrial dysfunction during and after the menopause transition and identify priorities for research focused on mitigating CVD risk in aging women.

**TABLE 1:** Methodological characteristics and principal findings of original studies within the last 10 years examining estrogen signaling, PGC‐1 regulation, and mitochondrial function.

Author study	Model/population	Estrogen manipulation	Exposure details	ER/signaling pathway	PGC‐1 outcome	Mitochondrial/cardiovascular outcome
Hamilton et al. (2016)	Aged, high‐fat‐fed OVX mice; cardiac and metabolic tissues	OVX with selective ERα agonist (PPT) versus OVX‐vehicle and sham‐vehicle	PPT 1.88 mg/kg/day by subcutaneous 21‐day‐release pellets during 12 weeks of Western high‐fat feeding	Selective ERα activation → PGC‐1α/NRF‐1 and mitochondrial gene expression	↑ cardiac PGC‐1α and NRF‐1 expression	Selective ERα activation improved mitochondrial respiration, insulin sensitivity, and adiposity
Park et al. (2017)	Early‐ and late‐postmenopausal women; transdermal estradiol	Estradiol versus placebo in early‐ and late‐postmenopausal women	Transdermal 17β‐estradiol, 0.15 mg for 1 week; randomized, blinded crossover; patches changed mid‐week	ERα/ERβ localization with AMPK phosphorylation and nuclear PGC‐1α signaling	↑ nuclear skeletal muscle PGC‐1α and pAMPK in early post‐menopause	Demonstrated timing‐dependent skeletal muscle responsiveness to estradiol
Jiang et al. (2022)	Vascular smooth muscle cells	ERα overexpression or knockdown in vascular smooth muscle cells exposed to simulated microgravity	In vitro genetic manipulation during simulated microgravity; no systemic estradiol treatment	ERα → NRF1 → OMI signaling controlling mitochondrial dynamics and mitophagy	— PGC‐1 not measured; ERα ↑ NRF1	Demonstrated ERα regulation of NRF1‐dependent mitochondrial quality control and mitophagy
Tian, Gao, et al. (2023)	OVX mice treated with 17β‐estradiol; liver	Bilateral OVX with 17β‐estradiol replacement versus OVX‐vehicle and sham controls	17β‐estradiol 20 μg/kg/day, subcutaneous, for 8 weeks	ERα → SIRT1‐mediated PGC‐1α deacetylation/activation	↑ hepatic PGC‐1α expression; ↓ PGC‐1α acetylation	Estradiol preserved mitochondrial function following ovarian hormone loss
Gowayed et al. (2024)	OVX female rats; cardiac tissue	Sham or OVX rats treated with estradiol, fulvestrant, both, or vehicle	17β‐estradiol 30 μg/kg and/or fulvestrant 5 mg/kg, subcutaneous daily for 28 days after 4‐week recovery	ER‐dependent and residual non‐receptor signaling involving AMPK, PGC‐1α/β, NRF1, and TFAM	E2 ↑ cardiac PGC‐1α and PGC‐1β expression; fulvestrant attenuated these effects	Receptor‐mediated estradiol treatment supported cardiac mitochondrial homeostasis
Robitaille et al. (2025)	Postmenopausal women undergoing cardiac surgery plus complementary mouse experiments	Human cardiac tissue comparison plus OVX/high‐fat‐fed mice with or without nicotinamide rescue	OVX at 8 weeks; 60%‐fat diet for 25 weeks; nicotinamide 400 mg/kg/day intraperitoneally for 7 days	NAD+/SIRT1 → AMPK phosphorylation → PGC‐1α signaling; estradiol was not replaced	↓ cardiac PGC‐1α and pAMPK in postmenopausal women and OVX/HFD mice; NAM ↑ both	Identified impaired cardiac mitochondrial signaling and evaluated nicotinamide rescue
Tian et al. (2025)	OVX mice with exercise intervention; skeletal muscle	OVX with endurance training versus sedentary OVX and sham controls	Bilateral OVX; treadmill training for 8 weeks beginning 10 weeks after surgery; no estradiol replacement	Exercise‐responsive AMPK/SIRT1/PGC‐1α signaling independent of myogenic estradiol synthesis	OVX ↓ skeletal muscle PGC‐1α; exercise or E2 ↑ PGC‐1α	Examined whether exercise preserves mitochondrial function independently of local estradiol synthesis

Abbreviations: E2, estradiol; OVX, ovariectomy; PPT, propyl pyrazole triol.

## 2 CONVERGENCE OF ESTROGEN SIGNALING AND PGC‐1 REGULATION

Estradiol is the predominant endogenous estrogen prior to menopause and exhibits the greatest affinity for estrogen receptors (ERs). However, among the major endogenous estrogens, estrone and estriol also bind ERs and promote downstream signaling (Paterni et al., 2014). Accordingly, in the following sections describing signaling mechanisms, the term “estrogen” will be used to reflect that these pathways may be activated by multiple estrogenic ligands.

At the cellular level, estrogen signals through both genomic and non‐genomic mechanisms mediated by nuclear and membrane‐associated ERα and ERβ, and G Protein‐Coupled Estrogen Receptor (GPER) (Figure 2) (Fuentes & Silveyra, 2019). These receptor pathways converge with the PGC‐1 family of transcriptional coactivators, master regulators of mitochondrial biogenesis and OXPHOS capacity (Kemper et al., 2014). Several studies in experimental models show that estradiol increases PGC‐1 coactivator activity through intracellular (i.e., genomic) and membrane‐associated (i.e., non‐genomic) ER signaling (Kemper et al., 2014; Tian, Hong, et al., 2023). There is also evidence, although to a lesser extent, that supplementing with estradiol similarly increases mRNA of PGC‐1α by 29% in young adult men (Maher et al., 2010) and nuclear PGC‐1α protein by 22% in early postmenopausal women (Park et al., 2017). Therefore, understanding how ER signaling regulates PGC‐1 protein expression and coactivator activity and how this relationship shifts across the female lifespan may therefore help identify strategies to preserve mitochondrial health during and after menopause.

**FIGURE 2:**
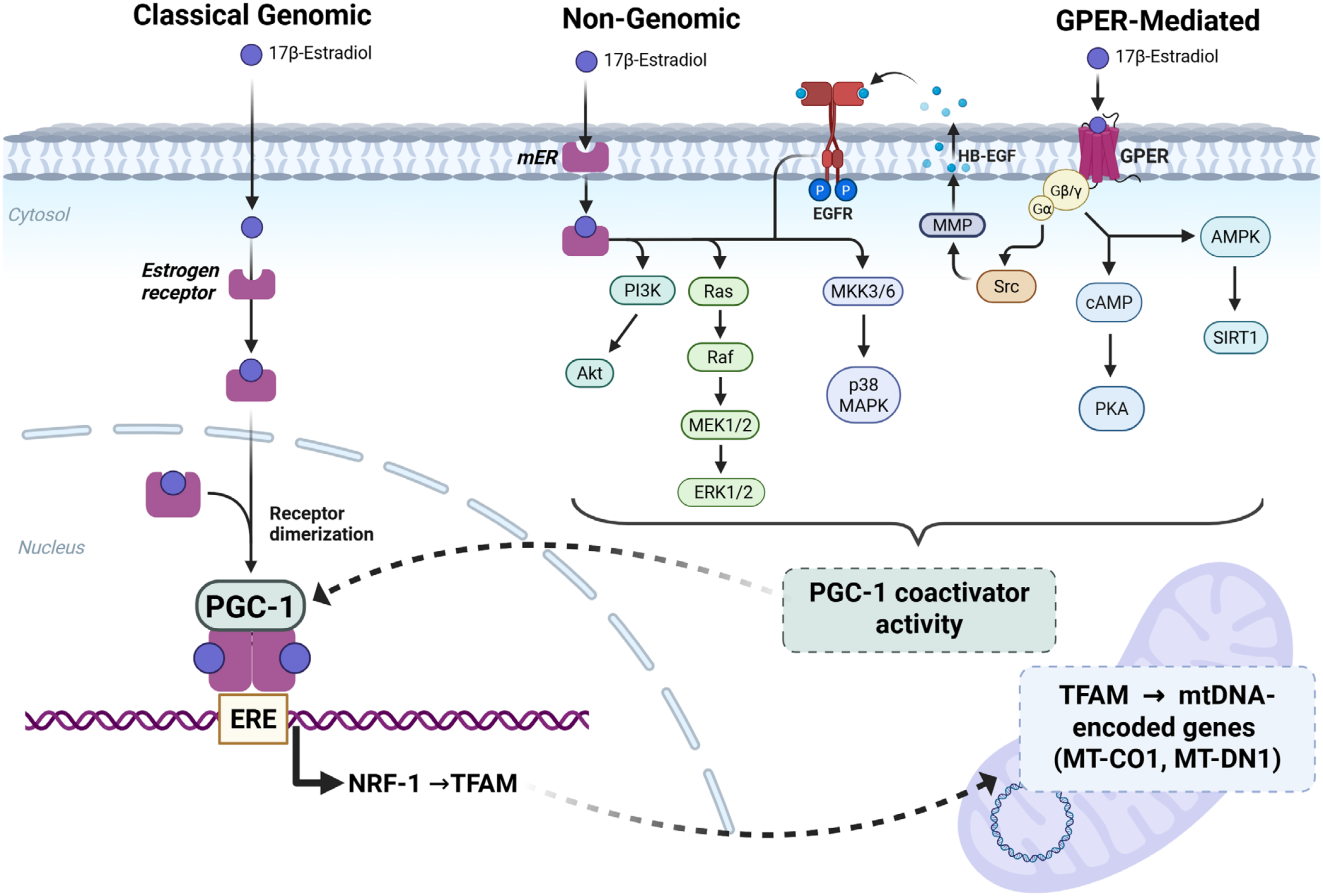
Genomic and non‐genomic estradiol signaling pathways regulating PGC‐1‐mediated mitochondrial function. *Classical genomic mechanism*: Estradiol diffuses across the cell membrane and binds intracellular estrogen receptors (ER), promoting receptor dimerization and translocation to the nucleus. In the classical paradigm, the ER‐complex recruits coactivators (CoA), including PGC‐1 isoforms, and binds estrogen response elements (ERE) on target genes to regulate transcription. Through PGC‐1 coactivation, ER signaling enhances expression of nuclear respiratory factor 1 (NRF‐1), which promotes transcription of mitochondrial genes and mitochondrial transcription factor A (TFAM). TFAM subsequently translocates to mitochondrial DNA (mtDNA) to regulate transcription of mtDNA‐encoded oxidative phosphorylation genes (e.g., MT‐CO1, MT‐ND1). *Non‐genomic mechanism*: In the non‐genomic mechanism, estradiol rapidly activates kinase signaling cascades through membrane‐associated ERs (mER). These pathways include PI3K‐Akt and MAPK signaling (e.g., ERK1/2 and p38 MAPK), which regulate cellular metabolism, survival signaling, and gene transcription, including the activation of PGC‐1 isoforms. *GPER‐mediated mechanism*: G‐protein‐coupled estrogen receptor (GPER) represents an additional membrane‐initiated, non‐genomic pathway through which estradiol exerts rapid effects. Upon activation, GPER stimulates dissociation of G‐protein subunits (Gα and Gβγ), initiating downstream signaling cascades including cAMP‐PKA and AMPK pathways. AMPK activation promotes increases in NAD^+^ availability, supporting SIRT1 activity and subsequent stimulation of PGC‐1. GPER signaling may also activate Src kinases, leading to matrix metalloproteinase (MMP)‐mediated cleavage of membrane‐bound heparin‐binding epidermal growth factor (HB‐EGF), resulting in transactivation of epidermal growth factor receptor (EGFR). EGFR activation converges with non‐genomic signaling pathways, including PI3K‐Akt and MAPK cascades, further contributing to the regulation of PGC‐1 activity.

## 3 CLASSICAL (DIRECT) GENOMIC REGULATION OF PGC‐1 BY ESTRADIOL

In the classical genomic pathway, estrogen diffuses across the plasma membrane and binds intracellular receptors, ERα or ERβ (Fuentes & Silveyra, 2019). With ovarian‐derived estradiol, genomic actions assist in the regulation of the female menstrual cycle, while estradiol administration promotes genomic actions within 3–6 h (Nilsson & Gustafsson, 2002; O'Lone et al., 2004). Ligand binding induces a conformational change in the receptor, promoting receptor activation and dimerization (Fuentes & Silveyra, 2019). Homo‐ or heterodimers (ERα/ERα, ERβ/ERβ, or ERα/ERβ) of ERs are then formed to bind estrogen response elements (ERE) located in the promoters or distal enhancer regions of target genes to regulate transcription (Ascenzi et al., 2006). Importantly, dimerization influences coactivator recruitment and activity, thereby influencing downstream gene expression (Ascenzi et al., 2006). Following activation and dimerization, the receptor complex translocates to the nucleus (if not already localized there) and directly binds EREs within regulatory regions of target genes (Ascenzi et al., 2006). PGC‐1α and PGC‐1β function as unique coactivators of ERs, with PGC‐1β particularly selectivity for ERα (Kressler et al., 2002, 2007). Their ligand‐dependent interaction involves the ER hinge domain, while their LXXLL motif (i.e., nuclear receptor box [NR‐box]) engages with the activation function‐2 (AF‐2) domain of the ER (Kressler et al., 2007). These protein–protein interactions thereby facilitate the assembly of transcriptional complexes at target‐gene promoters and promote transcription of estrogen‐responsive genes (Kressler et al., 2007). Among these transcriptional targets, estrogen enhances the expression of genes linked to mitochondrial biogenesis (e.g., NRF‐1, TFAM), mtDNA‐encoded respiratory genes (e.g., MTCO1/COI, MTND1/ND1), and nuclear‐encoded OXPHOS genes (e.g., COX IV, cytochrome c, ATP‐synthase genes), as well as antioxidant defense genes (e.g., SOD2/MnSOD) (Bjornstrom & Sjoberg, 2005). The resulting mRNA transcripts are translated into proteins that alter cellular phenotype, thereby contributing to cellular adaptations relevant to mitochondrial quality, content, and function.

## 4 TETHERED (INDIRECT) GENOMIC REGULATION OF PGC‐1 BY ESTRADIOL

Within the genomic functions of ER signaling is a non‐classical variant commonly referred to as the tethered estrogen signaling mechanism (Fuentes & Silveyra, 2019). Similar to the classical genomic pathway, tethered signaling is initiated by estrogen binding to intracellular ERα or ERβ (Fuentes & Silveyra, 2019). However, rather than directly binding EREs, ligand‐bound ERs interact with transcription factors already bound to DNA, including activator protein‐1 (AP‐1), nuclear factor kappa B (NF‐κB), and specificity protein 1 (Sp1) (Abu Shelbayeh et al., 2023; Bjornstrom & Sjoberg, 2005; Fuentes & Silveyra, 2019). These transcription factors regulate genes involved in cellular stress responses (AP‐1) (Shaulian & Karin, 2002), inflammatory pathways (NF‐κB) (Abu Shelbayeh et al., 2023), and basal and tissue‐specific transcriptional programs (Sp1) (O'connor et al., 2016). Tethered ER signaling may also be influenced by PGC‐1 coactivators through interactions between the PGC‐1 LXXLL motif and the ERα AF‐2 domain (Klinge, 2020). Although direct recruitment of PGC‐1 to tethered ER complexes has not been definitely established, there is evidence that PGC‐1α reciprocally regulates NF‐κB (Abu Shelbayeh et al., 2023). Future studies should examine a broader gene panel regulated by PGC‐1 coactivation of tethered ERs and determine how these targets may influence mitochondrial function.

## 5 RAPID NON‐GENOMIC REGULATION OF PGC‐1 BY ESTRADIOL

In addition to classical genomic regulation, estrogen also initiates rapid, non‐genomic cascades that generate mitochondrial responses within seconds to minutes (Klinge, 2020). Whereas genomic signaling is initiated by ligand binding to intracellular ERs, non‐genomic pathways are triggered at the plasma membrane, where estrogen binds membrane‐associated ERα or ERβ. These membrane‐localized receptors may consist of palmitoylated ERα or ERβ targeted to caveolae through interactions with scaffolding proteins such as caveolin‐1, facilitating rapid downstream signaling (Ascenzi et al., 2006; Fuentes & Silveyra, 2019).

Upon formation of the estrogen‐ER complex at the membrane, rapid activation of intracellular signaling cascades ensues. This often involves activation of the Src proto‐oncogene tyrosine kinase (Src) family of kinases and subsequent stimulation of various signaling pathways, including p38 mitogen‐activated protein kinase (p38 MAPK), a positive regulator of PGC‐1α expression and activity (Zhang et al., 2014), and phosphoinositide 3‐kinase/protein kinase B (PI3K/Akt), which promotes eNOS phosphorylation and NO production and regulates cellular glucose and lipid metabolism (Haynes et al., 2000; Li et al., 2007). PI3K/Akt also influences intracellular Ca^2+^ handling (Goetz et al., 1999), with elevated Ca^2+^ activating CaMK and calcineurin pathways that can promote PGC‐1α expression (Handschin et al., 2003; Zhang et al., 2014). Taken together, rapid membrane‐initiated signaling can influence PGC‐1 expression and activity through kinase‐ and calcium‐dependent pathways, promoting transcriptional programs relevant to mitochondrial function in the prevention and treatment of CVD (Chen et al., 2009; Klinge, 2020; Qian, Zhu, et al., 2024).

## 6 GPER‐MEDIATED REGULATION OF PGC‐1 BY ESTRADIOL

Predominantly localized intracellularly, GPER undergoes constitutive internalization from the plasma membrane, resulting in a receptor population that is largely observed within intracellular compartments while still remaining accessible to ligands such as estrogen (Cheng et al., 2011). Upon ligand binding and receptor activation, GPER initiates rapid cellular signaling beginning with the recruitment and activation of non‐receptor tyrosine kinases of the Src family that subsequently promotes GPER‐mediated epidermal growth factor receptor (EGFR) transactivation (Prossnitz & Barton, 2011). Src kinase signaling activates matrix metalloproteinases, releasing heparin‐binding epidermal growth factor (HB‐EGF) to activate EGFR and downstream signaling cascades (Prossnitz & Barton, 2011). Through this mechanism, GPER can activate several intracellular signaling pathways, including those described in the *Rapid Non‐Genomic Signaling* section, such as p38 MAPK, PI3K/Akt signaling, intracellular Ca^2+^ mobilization, as well as extracellular signal‐regulated kinase 1/2 (ERK1/2), AMP‐activated protein kinase (AMPK) and cyclic adenosine monophosphate/protein kinase a (cAMP/PKA) (Gowayed et al., 2024; Prossnitz & Barton, 2011). Activation of these pathways enables GPER to regulate PGC‐1α (p38 MAPK, AMPK), along with a variety of cellular processes that further support mitochondrial function and adaptation, including gene transcription (p38 MAPK, ERK1/2), cellular metabolism (AMPK, cAMP/PKA, PI3K/Akt), cell growth and survival (MAPK/ERK1/2, PI3K/Akt), hormonal regulation (cAMP/PKA), and calcium‐dependent contractile and signaling mechanisms (Ca^2+^ mobilization, with contributions from MAPK signaling) (Gowayed et al., 2024; Prossnitz & Barton, 2011). Notably, cellular energy status and hormones, including estrogen, can increase PGC‐1α activity through AMPK‐mediated phosphorylation and SIRT1‐mediated deacetylation (De Sousa, 2026; Tian, Gao, et al., 2023). GPER therefore provides an additional mechanism through which estrogen mediates rapid signaling pathways capable of regulating PGC‐1α protein expression and phosphorylation.

## 7 ESTRADIOL AND MITOCHONDRIAL REGULATION ACROSS THE FEMALE LIFESPAN

While the estradiol is traditionally discussed in the context of reproduction, its physiological reach affects a multitude of systems throughout the female lifespan. Although primarily produced in the ovaries, endogenous estradiol is also synthesized in several extra‐ovarian tissues, including the breast, brain, adipose tissue, and arterial wall, where it can exert localized physiological effects (Barakat et al., 2016). Circulating estradiol has systemic effects through the binding and activation of ERs, most prominently ERα and ERβ, which are complementarily expressed in highly metabolic tissues, such as skeletal muscle (Yoh et al., 2023), cardiac muscle (Qian, Liu, & Liu, et al., 2024), and vascular wall (Smith et al., 2026). From a cardioprotective perspective, estradiol exerts several metabolic, antioxidant, and vascular effects that produce favorable modification to circulating lipoproteins (Xin et al., 2025), glucose disposal and insulin sensitivity (Yan et al., 2019), and oxidative stress markers (Xiang et al., 2021). Furthermore, estradiol preserves vascular health by suppressing ROS production and enhancing antioxidant defenses, thereby limiting atherosclerotic remodeling while promoting angiogenesis and vasodilation (Orshal & Khalil, 2004; Xie et al., 2022). These actions are mediated through epigenetic and intracellular signaling cascades, such that changes in estradiol availability across the menopausal lifespan may alter pathways involved in mitochondrial function and cardiometabolic health (Edwards et al., 2026).

In murine models, estradiol deficiency following ovariectomy (OVX) reduces mitochondrial respiratory capacity and suppresses expression of key regulators of mitochondrial biogenesis (Hamilton et al., 2016; Tian, Hong, et al., 2023; Tian, Gao, et al., 2023). Conversely, chronic selective ERα activation in aged, high‐fat‐fed OVX mice increased cardiac PGC‐1α and NRF‐1 expression, enhanced mitochondrial respiratory function, and improved insulin sensitivity and visceral adiposity (Hamilton et al., 2016). In humans, however, the influence of sex‐hormone levels on skeletal muscle mitochondrial bioenergetics remains less definitive (Nelson et al., 2025; Ren et al., 2024). Ren et al. (2024) reported reduced phosphocreatine recovery and ATP generation during the early follicular (low‐hormone) phase compared to the late follicular (high‐estradiol) phase in pre‐menopausal women, suggesting estradiol modulates muscle bioenergetic capacity. In contrast, Nelson et al. (2025) observed no differences in maximal mitochondrial oxidative capacity or citrate synthase activity between early follicular and luteal phases, although subtle alterations in proton leak respiration and ROS emission were detected. Further, while basal substrate metabolism appears unchanged across the menstrual cycle, substrate use during exercise has been a point of contention in exercise science literature for decades. While some studies indicate reduced glucose dynamics and increased fat oxidation during aerobic exercise during high estradiol phases (Hulton et al., 2021), others show a lack of meaningful difference across various exercise intensities and hormone phases (Lofberg et al., 2025). Altogether, these findings suggest that while estradiol exerts clear mitochondrial and bioenergetic effects in experimental models, physiological concentrations of estradiol may produce more modest or context‐dependent responses in women.

The more profound physiological transition occurs when rhythmic hormone fluctuations cease at the onset of perimenopause. During the perimenopausal years, which begin on average 4–7 years before reaching menopause, estradiol fluctuations become increasingly erratic, with sporadic surges and precipitous declines (Elliott‐Sale et al., 2021). In combination with such changes in estradiol, luteal phase progesterone levels similarly become increasingly variable, contributing to disrupted cycle regularity and an increased occurrence of anovulatory cycles (i.e., absence of ovulation) (Hale et al., 2007; Harlow et al., 2012). Some data suggest that progesterone increases mitochondrial ROS emission regardless of estradiol availability, while also reducing mitochondrial respiration when estradiol levels are low (Kane et al., 2011). Estradiol is also well recognized for its role in regulating ROS production, with reduced concentrations increasing ROS and oxidative stress (Guajardo‐Correa et al., 2022). Thus, altered cyclical patterns of estradiol and progesterone during the menopause transition may further exacerbate oxidative stress and impair OXPHOS (Kane et al., 2011; Ventura‐Clapier et al., 2019). Another characteristic of irregular cycles as ovarian function declines is reduced ovarian negative feedback, resulting in progressively elevated follicle‐stimulating hormone (FSH) concentrations, even when ovulation does not occur consistently (Hale et al., 2007). Recently, FSH was shown to promote mitochondrial biogenesis through PGC‐1α deacetylation and activation in young female mice and cultured granulosa cells (Wu et al., 2026); however, it remains unclear how progressively elevated FSH during the menopausal transition influences this signaling and mitochondrial responses in women.

As perimenopause progresses, irregular and anovulatory cycles become more prevalent and are associated with vasomotor, somatic, and psycho‐emotional symptoms (Thurston & Joffe, 2011). Vasomotor symptoms, particularly hot flushes and night sweats, are among the most common perimenopausal symptoms and affect quality of life in up to 80% of women (Franco et al., 2015). Estradiol exerts several beneficial effects on vascular function, of which include NO‐mediated vasodilation (Guajardo‐Correa et al., 2022). Thus, sporadic estradiol fluctuations during perimenopause may contribute to dysregulated eNOS/NO signaling, reduced vasodilation, and impaired heat dissipation (Guajardo‐Correa et al., 2022; Moreau et al., 2024; Szmuilowicz et al., 2011). Interestingly, late‐onset vasomotor symptoms are associated with greater CVD risk (Szmuilowicz et al., 2011), with frequent and persistent vasomotor symptoms linked to a 77% higher risk over ~22 years of follow‐up (Thurston & Joffe, 2011).

Once a woman experiences 12 consecutive months without a menses (typically in her early 50s) she is classified as postmenopausal. This transition is marked by a sustained decline in circulating estradiol and a shift toward estrone predominance, a weaker estrogen with lower affinity for ERs (Elliott‐Sale et al., 2021). Estradiol deficiency has been associated with impairments in mitochondrial and metabolic function, while hormone replacement has been investigated as a potential means to restore these outcomes (Kleis‐Olsen et al., 2024). Consistent with this, estradiol treatment in early menopausal women has been shown to improve metabolic function, even in the presence of advancing age and markers of metabolic syndrome (Kleis‐Olsen et al., 2024; Park et al., 2017; Pereira et al., 2015) When comparing hormone therapy users to non‐users, long‐term combined estradiol‐progestin therapy has been associated with greater skeletal muscle mitochondrial content and respiratory capacity, as well as lower visceral and total fat mass (Kleis‐Olsen et al., 2024). In addition, short‐term, high‐dose transdermal estradiol administration has been shown to enhance skeletal muscle nuclear ERα and ERβ content, PGC‐1α abundance, and AMPK phosphorylation in early, but not late, postmenopausal women (Park et al., 2017). The altered estrogen milieu in peri‐ and post‐menopausal women coincides with emerging evidence of reduced PGC‐1α protein expression (Park et al., 2017; Robitaille et al., 2025), mitochondrial dysfunction (Ventura‐Clapier et al., 2019), impaired vascular function, and increased visceral fat accumulation in aging women (Mosca et al., 2007; Qian, Liu, & Liu, 2024; Ventura‐Clapier et al., 2019). Such temporal alignment is consistent with epidemiological evidence demonstrating that cardiovascular and metabolic risk accelerate following menopause (El Khoudary et al., 2020; Mosca et al., 2007).

Although estradiol treatment has been shown to improve menopause‐related symptoms and disease outcomes (Kleis‐Olsen et al., 2024; Park et al., 2017; Sarri et al., 2017), concerns regarding hormone therapy persist due to earlier studies linking its use to adverse effects, particularly following the Women's Health Initiative (WHI) trial (Rossouw et al., 2002). Estradiol replacement was first introduced in the mid‐20th century but following the WHI study in 2002, a black box warning was issued for hormone therapy due to increased risks of breast cancer, stroke, coronary heart disease, and venous thromboembolism (Rossouw et al., 2002). After which, the use of menopausal hormone therapy dropped by 80% in the United States (Crawford et al., 2019). More recently, a longitudinal cohort study of 876,805 women reported no increased risk of all‐cause mortality with menopausal hormone therapy use (Mikkelsen et al., 2026). The discrepancy between the WHI study and recent longitudinal study by Mikkelsen et al. (2026) is likely due to the age and timing of hormone therapy use, as the WHI participants were 10 years or more post‐menopause with an average age of 63 and many with existing cardiometabolic diagnoses. These findings are also consistent with Park et al. (2017), who reported that women more than 6 years post‐menopause exhibited diminished responsiveness to estradiol treatment compared to women earlier in the menopausal transition. Despite these recent reports, perceptions of hormone therapy safety remain influenced by earlier evidence and may be slow to shift. Thus, alternative strategies should continue to be explored as strategies to enhance PGC‐1 signaling and mitigate menopause‐related symptoms and cardiometabolic decline in women.

## 8 CELL‐SPECIFIC CONVERGENCE OF ESTRADIOL, PGC‐1 SIGNALING, AND CARDIOVASCULAR FUNCTION

Current evidence demonstrates that estrogen signaling enhances cellular and mitochondrial function in a cell‐ and tissue‐specific manner in humans and rodents, with differences stemming from ER isoform expression, subcellular localization, and coregulator recruitment (Kim et al., 2014). ERα and ERβ concentrations vary among cardiomyocytes, vascular endothelial cells, and vascular smooth muscle cells, with specific ERα isoforms appearing to mediate many of estradiol's cardioprotective effects (Jiang et al., 2022; Kim et al., 2014; Pugach et al., 2016). The most prevalent ERα subunits expressed in cardiomyocytes and vascular endothelial cells are ERα66 (full‐length ERα) and ERα46 (short‐form ERα), respectively (Kim et al., 2014; Pugach et al., 2016). ERα expression in human myocardium does not appear to significantly differ between pre‐ and postmenopausal women (Mahmoodzadeh et al., 2014), although reduced cardiomyocyte estradiol signaling is associated with increased stress‐related cardiomyocyte hypertrophy from impaired mitochondrial fission and mitophagy processes, and more prominent tissue and mitochondrial damage follow cardiac injury (Calle et al., 2025; Robitaille et al., 2025). In contrast, endothelial ERα expression is approximately 33% lower in postmenopausal women than in premenopausal during the late follicular phase and is associated with reduced vasodilatory capacity and greater susceptibility to atherosclerotic vascular dysfunction (Gavin et al., 2009; Svedberg et al., 2026). Taken together, cell‐specific changes in estradiol signaling and ER expression may alter estradiol‐PGC‐1 interactions, with declining estradiol across the menopause transition potentially disrupting mitochondrial and cardiovascular function.

Prior to perimenopause, physiological levels of estradiol contribute to the maintenance of cardiomyocyte and vascular endothelial cell function (Calle et al., 2025; Moreau et al., 2024). These protective effects are mediated in part through ERα‐dependent signaling, with ERα protein expression and cellular responsiveness varying by sex, age, and hormonal status (Mahmoodzadeh et al., 2014; Moreau et al., 2020). Estradiol‐ERα signaling in cardiomyocytes and endothelial cells has been proposed as one mechanism contributing to the lower cardiovascular and metabolic disease risk observed in pre‐menopausal females relative to age‐matched males (Moreau et al., 2020; Quiroga et al., 2024). Consistent with this, female cardiomyocytes exhibit greater mitochondrial resilience than males, characterized by more efficient respiratory chain organization, lower mitochondrial ROS, and improved Ca^2+^ handling (Clements et al., 2023); greater preservation of mitochondrial membrane potential and bioenergetic capacity under stress (Scott et al., 2022), and more precise ATP production through enhanced sarcoplasmic reticulum‐mitochondrial coupling (Rhana et al., 2026). Although direct measurements of cardiomyocyte and endothelial estradiol‐ERα signaling across perimenopause remain limited, endothelial function begins to decline markedly during late perimenopause, coinciding with reductions in estradiol, and impaired cardiac function and remodeling become more evident following menopause (Moreau et al., 2020; Sung et al., 2022). These observations suggest that declines in circulating estradiol and altered ERα expression in cardiomyocytes and endothelial cells across and following the menopause transition may contribute to reduced PGC‐1‐mediated mitochondrial function and the progressive loss of cardiovascular protection (Figure 3).

**FIGURE 3:**
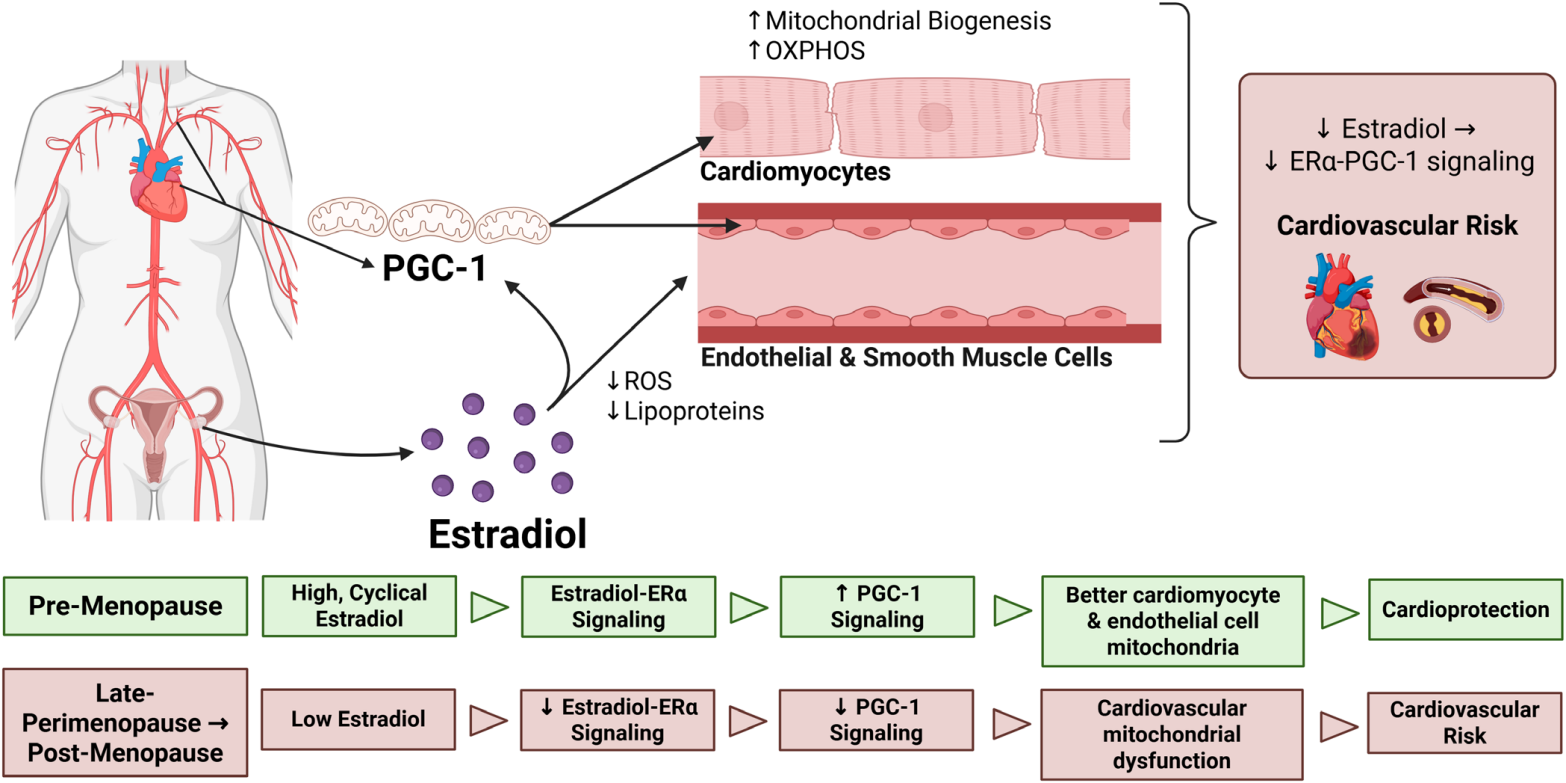
Estradiol‐PGC‐1 signaling on cardiovascular mitochondrial function and disease risk across the menopausal transition. Prior to menopause, high, cyclical estradiol concentrations support ERα‐mediated PGC‐1 signaling within cardiovascular tissues (i.e., cardiomyocytes, endothelial cells, and vascular smooth muscle cells). Increased PGC‐1 signaling promotes mitochondrial biogenesis and OXPHOS in cardiomyocytes and the vascular wall while reducing ROS production within these tissues. Endogenous estradiol produced by the ovaries can act within the cardiovascular system to promote these processes through PGC‐1 coactivation of ERα (genomic mechanism) and kinase cascades that increase PGC‐1 protein expression and coactivator activity (non‐genomic mechanism). Together, PGC‐1 and estradiol‐ERα signaling contribute to improved cardiovascular mitochondrial function and cardioprotection. Declining estradiol across the menopausal transition may reduce this signaling, impairing mitochondrial biogenesis, OXPHOS, and redox regulation. The convergence of these pathways represents one mechanism through which the progressive loss of estradiol may influence cardiovascular health.

Full‐length ERα66 is the predominant ERα isoform in neonatal rat ventricular myocytes (NRVMs) and is primarily localized to the nucleus, consistent with a prominent role in classical genomic estradiol signaling in cardiac muscle (Pugach et al., 2016). ERα66 has also been identified in human tissues, although its relative abundance in human cardiomyocytes is less clear (Irsik et al., 2013). In mouse models of heart failure, the PGC‐1α promoter contains an ERα‐binding site within its first 600 base pairs, with mitochondrial gene expression remaining higher in female than male models of heart failure (Ruiz et al., 2012; Witt et al., 2008). Importantly, Witt et al. (2008) demonstrated that estradiol alone did not significantly activate the PGC‐1α promoter, whereas estradiol in the presence of ERα significantly increased PGC‐1α promoter activity. Following intracellular activation and translocation to the nucleus, the LXXLL motif of PGC‐1α can interact with the AF‐2 domain of ERα, allowing PGC‐1α to potentiate ERα‐dependent transcription of mitochondrial gene programs involved in mitochondrial biogenesis (Kressler et al., 2002). PGC‐1α also strongly interacts with transcriptional regulators, ERRα and ERRγ, within cardiomyocytes to regulate genes controlling fatty‐acid oxidation, mitochondrial respiration, OXPHOS, and ATP‐producing capacity (Zhou et al., 2021). Consistent with these mechanisms, PGC‐1α expression was maintained in female mouse models of heart failure and restored following selective ERα activation in metabolically compromised, estradiol‐deficient female mice (Hamilton et al., 2016; Witt et al., 2008). These mechanistic insights further support the cardioprotective effects of estradiol‐PGC‐1α interactions against CVD and heart failure while also emphasizing the importance of not simply higher estradiol levels, but sufficient ERα expression within cardiomyocytes to support nuclear genomic signaling.

Human endothelial cells are abundant in a truncated short‐form ERα (i.e., ERα46), which efficiently mediates membrane‐initiated non‐genomic signaling underlying rapid vascular responses (Haynes et al., 2003; Kim et al., 2014; Li et al., 2003; Russell et al., 2000). Membrane‐associated ERα signaling activates PI3K/Akt and MAPK, which are particularly relevant to vascular endothelial and smooth muscle cell function because both promote eNOS/NO signaling and subsequent dilatory changes. Through PI3K/Akt, estradiol promotes phosphorylation and activation of eNOS and increases NO production and bioavailability, thereby supporting vasodilation and vascular tone (Haynes et al., 2000, 2003), as well as anti‐inflammatory and anti‐atherogenic signaling (Borrás et al., 2010; Kemper et al., 2014; Villablanca et al., 2009). Similarly, data indicate that in vascular endothelial cells, estradiol rapidly activates MAPK through non‐genomic mechanisms, further supporting eNOS/NO‐signaling and associated vascular smooth muscle responses (Russell et al., 2000).

GPER‐mediated signaling also engages PI3K/Akt and MAPK, as well as cellular energy‐sensor AMPK (Fuentes & Silveyra, 2019; Prossnitz & Barton, 2011). Evidence from cardiac and hepatic models further suggests that estradiol can engage AMPK and ERα/SIRT1/PGC‐1α signaling, respectively, to support mitochondrial regulation (Gowayed et al., 2024; Tian, Hong, et al., 2023). Mechanistically, AMPK can activate PGC‐1α through phosphorylation, while SIRT1‐mediated deacetylation further increases PGC‐1α activity and mitochondrial regulatory capacity (Gowayed et al., 2024; Tian, Hong, et al., 2023). However, the extent to which ERα/AMPK/SIRT1/PGC‐1 signaling specifically mediates estradiol‐dependent mitochondrial adaptations in vascular endothelial and smooth muscle cells remains less clearly defined. In addition to AMPK‐mediated regulation of PGC‐1α, MAPK and PI3K/Akt signaling have also been implicated in the regulation of PGC‐1 expression and activity (Li et al., 2007; Zhang et al., 2014). Together, the convergence of estradiol‐PGC‐1 pathways in cardiac and vascular cells supports their role in mitochondrial function, highlighting a potentially important estradiol‐PGC‐1 link in mitigating peri‐ and post‐menopausal cardiomyocyte and endothelial decline and ultimately cardiovascular disease risk.

## 9 CONCLUSIONS AND IMPLICATIONS FOR MITOCHONDRIAL AND CARDIOVASCULAR HEALTH

The present review highlights the mechanistic intersections between estradiol signaling and PGC‐1 coactivator activity and proposes that changes in estradiol bioavailability and ER expression may influence cardiovascular mitochondrial quality and health. The available evidence supports the following inferences: (1) Estradiol may contribute to mitochondrial function through genomic and non‐genomic mechanisms that enhance PGC‐1 isoform expression and activity; (2) the progressive decline in estradiol concentrations during the menopausal transition may contribute to impairments in mitochondrial function through reductions in pathways that support PGC‐1 signaling; and (3) late perimenopause through early post‐menopause represents a critical physiological transition marked by low estradiol availability that may increase cardiovascular risk in women, in part through reductions in PGC‐1‐mediated mitochondrial function.

These observations underscore the importance of identifying the mechanisms underlying cardiovascular decline in women, particularly those exacerbated by estradiol loss. Given the central role of PGC‐1 signaling in mitochondrial function and emerging evidence supporting estradiol's role in maintaining PGC‐1 protein expression and coactivator activity, it warrants further investigation. Future studies should determine the extent to which estradiol loss alters PGC‐1‐mediated cardiovascular pathways, whether these effects are isoform‐specific in cardiac and vascular tissues, and whether hormonal or non‐hormonal strategies can preserve PGC‐1 content and function despite declining estradiol.

## AUTHOR CONTRIBUTIONS

**Alyssa L. Parten:** Conceptualization; visualization. **Glenn C. Rowe:** Writing – review and editing; supervision; conceptualization.

## FUNDING INFORMATION

This work was supported in part by grants from the National Institute of Health (NIH) T32 Postdoctoral Trainee (HL007457) to A.L.P. and (DK121299 and AG069906) to G.C.R.

## CONFLICT OF INTEREST STATEMENT

None of the authors has any conflict of interest in the submission of this manuscript.

## ETHICS STATEMENT

This article is a narrative review of previously published research and does not involve original research with human participants or animals. Therefore, institutional ethics approval and informed consent were not required.

## Data Availability

Data sharing is not applicable to this article because no new data were created or analyzed in this study.
